# ST-Elevation Myocardial Infarction in the Presence of Septic Shock

**DOI:** 10.1155/2020/8879878

**Published:** 2020-08-18

**Authors:** Leah Ishmael, Joseph Zalocha

**Affiliations:** HCA Healthcare/USF Morsani College of Medicine GME Programs, Northside Hospital, St. Petersburg, FL, USA

## Abstract

Elevated cardiac enzymes are often seen in the setting of sepsis. The mechanism involves hypoperfusion and possible compromise to myocardial tissue. Electrocardiogram (ECG) changes in the setting of septic shock are less common and can vary widely. Rarely, ST-segment elevations can occur. This case describes a 54-year-old female who presented with septic shock secondary to pyelonephritis and *Escherichia coli* bacteremia. The patient was admitted to the intensive care unit on norepinephrine and required mechanical ventilation. A significant rise in troponin I (peak 19.8 ng/mL) was seen and ECG showed ST-segment elevations in leads I and aVL with reciprocal ST depressions in leads II, III, and aVF. The patient was taken urgently for left cardiac catheterization, which showed no evidence of obstructive coronary artery disease. When distinguishing between septic shock and cardiogenic shock, insertion of a pulmonary artery catheter may help with diagnosis and treatment of cardiogenic shock. Catheter hemodynamic monitoring can also confirm the diagnosis. In our patient's case, hemodynamic monitoring was initiated and was not consistent with cardiogenic shock. ST-segment elevations in the high lateral leads and elevated cardiac markers were likely due to severe transmural ischemia secondary to increased oxygen demand. The patient was continued on intravenous antibiotics for treatment of her septic shock. She was extubated and weaned off of norepinephrine within 48 hours. Repeat ECG performed after resolution of the infection showed normal sinus rhythm with no ST-segment changes. Cardiac dysfunction in the setting of septic shock is well described in medical literature; however, the mechanisms of dysfunction are not explicitly understood. Transient hypoperfusion, coronary vasospasm, and localized endothelial damage are possible components. It is important to think of varying etiologies, other than acute coronary syndrome when approaching patients in septic shock with acute ST-segment changes and elevated cardiac markers.

## 1. Introduction

Elevated cardiac enzymes, specifically troponin I, are commonly seen in acute coronary syndromes (ACS), however can also occur in the setting of sepsis. The mechanism of elevated troponin in sepsis involves hypoperfusion or can also be the result of the infection spreading to cardiac tissue. ECG changes in the setting of septic shock are a less common finding, however do occasionally occur. Some associated ECG changes observed in the setting of sepsis include prolonged QTc interval, Osborn waves, bundle branch blocks, nonspecific ST changes, and very rarely ST-segment elevations [1]. The exact mechanism of ST-segment elevation seen in sepsis is not well understood.

## 2. Case Report

A 54-year-old female with a past medical history of diabetes mellitus and recurrent urinary tract infections presented for the evaluation of abdominal pain, fever, and dysuria. The patient had been experiencing these symptoms for three days. Her abdominal pain was mainly located in the right lower quadrant and right flank. It was described as intermittent and dull in sensation. She had been taking acetaminophen and ibuprofen at home with minimal relief of her symptoms. She was noted to be increasingly lethargic, prompting her daughter to bring her to the emergency department for further evaluation.

She has a history of insulin-dependent diabetes mellitus and has been noncompliant with her home insulin for the past three months. She has a history of multiple urinary tract infections that usually resolve on their own or after a course of antibiotics. At the time of presentation, she denied taking any home medications and reported no drug allergies. Family history is significant for heart disease in her mother. She has no history of prior surgeries and has a 30-pack year smoking history. She denies alcohol and recreational drug use.

Upon arrival to the emergency department (ED), the patient was minimally responsive and obtunded, requiring intubation for airway protection.

## 3. Hospital Course

**Figure 1:**
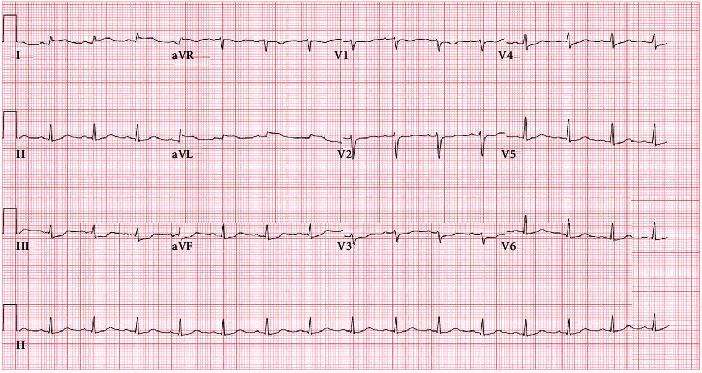
ECG with evidence of ST-segment elevations in leads I and aVL and reciprocal ST depressions in leads II, III, and aVF, consistent with lateral STEMI.

**Figure 2:**
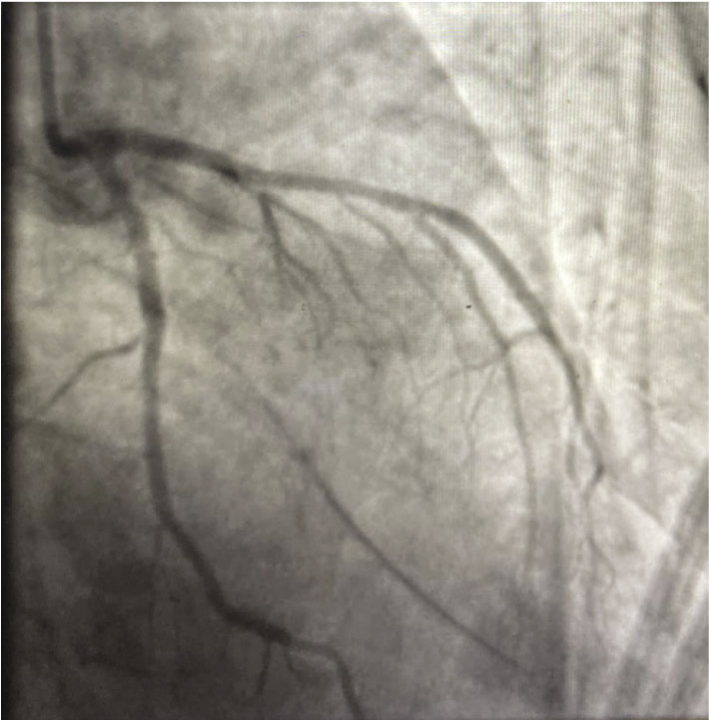
Coronary angiography demonstrating patent left coronary circulation.

The patient was admitted to the intensive care unit for further management. She was initially hypotensive upon admission with a blood pressure of 89/55 mmHg. Temperature was 97.8 degrees Fahrenheit, heart rate was 107 beats per minute, respiratory rate was 18 breaths per minute, and oxygen saturation was initially 89% on room air. On physical exam, the patient was intubated and minimally responsive off of sedation. Her pupils were equal and reactive to light. She met sepsis criteria with an initial white blood cell count (WBC) of 18.48 × 10^3^/*μ*L and tachycardia. She was started on intravenous antibiotics and given the sepsis fluid bolus. Her blood pressure did not improve after receiving fluids and she was started on norepinephrine to maintain a mean arterial pressure between 60 and 65 mmHg. Blood and urine cultures were obtained and were positive for *Escherichia coli*. Computed tomography (CT) scan of the abdomen/pelvis without contrast was performed and showed mild right hydroureter and hydronephrosis with no evidence of obstruction and perinephric inflammatory changes. Blood urea nitrogen (BUN) and creatinine were elevated at 73 mg/dL and 3.36 mg/dL, respectively. Troponins were ordered upon arrival and were initially negative, however trended upward to 0.02 ng/mL, 1.46 ng/mL, and 19.8 ng/mL, respectively. Initial ECG performed in the ED upon arrival showed sinus tachycardia with no acute ischemic changes. A repeat ECG was performed with the first elevated troponin and showed signs of minimal ST elevations in leads I and aVL. ECG performed with the third elevated troponin showed ST elevations in leads I and aVL with reciprocal ST depressions in leads II, III, and aVF (Figure 1). Central venous oxygen saturation (ScvO2) monitoring was initiated and initial value was 75%. The patient was taken for emergent catheterization for suspected ST-elevation myocardial infarction (STEMI). Left heart catheterization showed no evidence of obstructive coronary artery disease (Figure 2). Two-dimensional (2D) echocardiogram was performed which showed mildly reduced systolic function with an ejection fraction of 45% and mild diffuse hypokinesis. The patient was continued on intravenous antibiotic treatment for septic shock secondary to pyelonephritis and *Escherichia coli* bacteremia. She was eventually extubated and weaned off of norepinephrine.

## 4. Discussion

In the presence of ST-segment elevations, elevated troponins, and systemic hypotension, cardiogenic shock is high on the differential diagnosis list. When cardiogenic shock is suspected, diagnostic evaluation should be performed along with resuscitative efforts [2]. Echocardiogram should be performed immediately and can confirm the diagnosis. Findings on echocardiography specific to cardiogenic shock include severely depressed left and/or right ventricular systolic function, elevated filling pressures, and decreased stroke volume. Echocardiogram can also detect other causes of cardiogenic shock including cardiac tamponade, severe valvular abnormalities, and ventricular septal rupture [3]. In this patient, echocardiogram findings showed only mild diffuse hypokinesis and no evidence of pericardial effusion, valvular abnormalities, or elevated filling pressures. Echocardiographic studies suggest that 40-50% of patients with prolonged septic shock develop myocardial depression [4].

Hemodynamic monitoring can also help to determine the underlying etiology of shock. Measurement of central venous pressure and central venous oxygenation can help guide resuscitation in the management of shock. Placement of a pulmonary artery catheter can confirm the diagnosis of cardiogenic shock and help guide management. In this patient, ScvO2 was 75%, making the diagnosis of cardiogenic shock less likely [5]. In the event that this was cardiogenic shock secondary to acute myocardial infarction, management would still be focused on hemodynamic support. Norepinephrine is the initial vasopressor of choice. Use of an intra-aortic balloon pump is still controversial; however, benefit may exist in patients who are rapidly deteriorating or who have mechanical defects, such as mitral regurgitation. Other mechanical devices, such as left ventricular assist devices (LVAD) are increasingly being used, though evidence of improved outcome is lacking [6].

In this patient, ST-segment elevation in the high lateral leads and elevated troponins were likely due to severe transmural ischemia secondary to increased oxygen demand. Apart from ischemia, several other factors may have contributed to the microinjury and myocardial cell damage in the setting of septic shock. Endotoxins, cytokines, and reactive oxygen radicals induced by the infectious process may also have had a direct myocytotoxic effect [7].

The patient completed her intravenous antibiotic course for treatment of her septic shock secondary to pyelonephritis and *Escherichia coli* bacteremia. Repeat ECG was performed after resolution of the infection and showed normal sinus rhythm with no ST-segment changes. The patient also had a repeat echocardiogram performed that showed an EF of 60-65% and no regional wall motion abnormalities. She was started on daily aspirin and atorvastatin for risk factor modification.

## 5. Conclusion

Septic shock, by definition, entails end organ damage. In this patient, the presence of ST-segment elevations in the high lateral leads was likely secondary to ischemia and increased oxygen demand. Coronary angiography aided in this patient's diagnosis. However, due to the hemodynamic instability usually seen in septic shock patients, emergent angiography often cannot be performed. Clinicians should be aware of similar cases and implement interventions, such as pulmonary artery catheter placement and catheter hemodynamic monitoring, to aid in diagnosis and management.

## Data Availability

Data sharing not applicable to this study as no datasets were generated or analyzed during the current study.
